# Activation of the unfolded protein response in sarcoma cells treated with rapamycin or temsirolimus

**DOI:** 10.1371/journal.pone.0185089

**Published:** 2017-09-19

**Authors:** Joseph W. Briggs, Ling Ren, Kristi R. Chakrabarti, Yien Che Tsai, Allan M. Weissman, Ryan J. Hansen, Daniel L. Gustafson, Yousuf A. Khan, Jonathan D. Dinman, Chand Khanna

**Affiliations:** 1 Tumor Metastasis Biology Section, Pediatric Oncology Branch, Center for Cancer Research, National Cancer Institute, National Institutes of Health, Bethesda, Maryland, United States of America; 2 Laboratory of Protein Dynamics and Signaling, Center for Cancer Research, National Cancer Institute, National Institutes of Health, Frederick, Maryland, United States of America; 3 Colorado State University Flint Animal Cancer Center, Fort Collins, Colorado, United States of America; 4 Department of Cell Biology and Molecular Genetics, University of Maryland, College Park, Maryland, United States of America; University of Hong Kong, HONG KONG

## Abstract

Activation of the unfolded protein response (UPR) in eukaryotic cells represents an evolutionarily conserved response to physiological stress. Here, we report that the mTOR inhibitors rapamycin (sirolimus) and structurally related temsirolimus are capable of inducing UPR in sarcoma cells. However, this effect appears to be distinct from the classical role for these drugs as mTOR inhibitors. Instead, we detected these compounds to be associated with ribosomes isolated from treated cells. Specifically, temsirolimus treatment resulted in protection from chemical modification of several rRNA residues previously shown to bind rapamycin in prokaryotic cells. As an application for these findings, we demonstrate maximum tumor cell growth inhibition occurring only at doses which induce UPR and which have been shown to be safely achieved in human patients. These results are significant because they challenge the paradigm for the use of these drugs as anticancer agents and reveal a connection to UPR, a conserved biological response that has been implicated in tumor growth and response to therapy. As a result, eIF2 alpha phosphorylation and Xbp-1 splicing may serve as useful biomarkers of treatment response in future clinical trials using rapamycin and rapalogs.

## Introduction

The unfolded protein response is an evolutionarily conserved mechanism to respond to alterations in cellular homeostasis including endoplasmic reticulum (ER) stress [1, 2]. For example, conditions which promote the accumulation of unfolded proteins results in activation of the resident endoplasmic reticulum (ER) protein IRE1 alpha (inositol requiring enzyme-1 alpha). Following trans-autophosphorylation and dimerization of the luminal domain, IRE1 demonstrates a unique endonuclease activity in its cytoplasmic domain resulting in unconventional splicing of Xbp-1 (X-box binding protein 1) mRNA [3–5]. Xbp-1 is the only known mRNA substrate which undergoes such cleavage by IRE-1 and therefore serves as a specific biomarker for UPR induction [6]. The spliced form of Xbp-1 results in a frameshift in the amino acid sequence whereby the resulting protein is converted into a potent transcriptional activator [5]. Initiating transcription of Xbp-1 target genes such as BiP (binding immunoglobulin protein)/Grp78 (glucose regulated protein 78 kDa) and CHOP (C/EBP homologous protein) is a key event in UPR induction [7–11]. For example, upregulation of the chaperone, BiP/Grp78, increases the protein folding capacity of the ER and regulates gating of the ER translocon pore to maintain homeostatic calcium levels [12, 13]. However, under chronic ER stress conditions, sustained transcription of CHOP can result in growth arrest or apoptosis [14].

A second feature of the UPR, shared with the integrated stress response (ISR), is phosphorylation of eIF2 alpha at serine 51[15]. When unfolded proteins accumulate within the ER lumen, the chaperone BiP is sequestered away from the ER resident kinase PERK (protein kinase RNA PKR-like ER kinase) resulting in PERK activation [16]. Kinase activity in the PERK cytoplasmic domain phosphorylates eIF2a (Ser51) inhibiting translation initiation leading to a decrease in protein synthesis. Together, these UPR signaling events determine cell fate in response to acute and chronic stress.

Rapamycin and structurally related analogs (rapalogs) belong to a class of macrolide compounds recently approved and currently being evaluated in clinical trials to treat many types of cancer [17, 18]. However, the promise of these drugs is tempered by the fact that clinical responses, especially in solid tumors, have been infrequent, sporadic and not predicted by pharmacodynamic biomarkers. The best characterized mechanism of action for rapamycin/raplogs involves inhibition of mammalian target of rapamycin (mTOR) protein kinase activity. This occurs through binding of the immunophilin protein FKBP12/rapamycin complex to the FRB domain of mTOR [19, 20]. However, a previous study suggested that the potent growth inhibitory effects on cancer cells may not occur through this canonical mechanism of action and might involve direct binding of temsirolimus to mTOR leading to more robust inhibition of mTOR activity [20]. This previous study showed that micromolar exposures were required to inhibit total protein synthesis and resulted in other molecular changes consistent with ER stress such as phosphorylation of eIF2 alpha (Ser51) and increased steady-state levels of ATF4 protein. However, it was unclear whether these effects were due to activation of the integrated stress response or, more specifically, activation of the unfolded protein response.

Our data confirms that micromolar exposures of temsirolimus also provides maximal growth inhibition of osteosarcoma and rhabdomyosarcoma cell lines and results in inhibition of protein synthesis and phosphorylation of eIF2a (Ser51). However, we provide additional data demonstrating splicing of Xbp1, which is specific to activation of the unfolded protein response. This is supported by increased expression of Xbp1 target genes such as BiP and CHOP. In addition, we also demonstrate that the parent compound, rapamycin, induces Xbp1 splicing.

Similar to the findings of Shor et al, co-treatment with FK506, which competes with temsirolimus for FKBP12 binding, abrogated only low dose, but not high dose temsirolimus mediated growth inhibitory effects in sarcoma cells. However, our data suggests UPR induction by rapamycin/rapalogs may not simply be the result of more complete mTOR inhibition, since treatment with structurally unrelated, small molecule ATP competitive inhibitors of mTORC1/2, did not induce Xbp1 splicing. Therefore, our data argues against a direct role for mTOR in mediating the high dose effects of rapamycin/rapalogs.

Finally, we were able to detect temsirolimus in ribosome fractions in which 28S rRNA nucleotides were protected from chemical modification consistent with direct interaction of the drug with ribosomes within the peptide exit tunnel. We propose that this may alter co-translational folding of nascent peptides thereby activating the unfolded protein response.

Taken together, our data supports the idea that UPR unduction, following low micromolar exposures of rapamycin/rapalogs, is linked to some of their anticancer activity. These findings should aid in the rational selection of treatment doses and schedules and may also enable the selection of more relevant pharmacodynamics biomarkers, such as spliced Xbp1 mRNA and eIF2a (Ser51) protein phosphorylation, to guide therapeutic decisions.

## Results and discussion

### Treatment with temsirolimus or rapamycin at low micromolar exposures induces Xbp-1 splicing and transcription of UPR genes

First, we sought to determine the extent to which rapamycin and the rapalog, temsirolimus, could activate a sustained unfolded protein response in tumor cells. The most sensitive and specific molecular marker for UPR induction is the unconventional splicing of Xbp-1 mRNA. Osteosarcoma (143B and HOS-MNNG) and rhabdomyosarcoma (Rh30 and RD) cells were included in this study because they represent tumor cell types previously shown to be responsive to rapamycin and rapalogs[21–23]. Furthermore, mTOR was first isolated from human osteosarcoma cells as a result of studies to determine the protein(s) responsible for cell cycle inhibition in response to rapamycin [19, 24].

**Fig 1:**
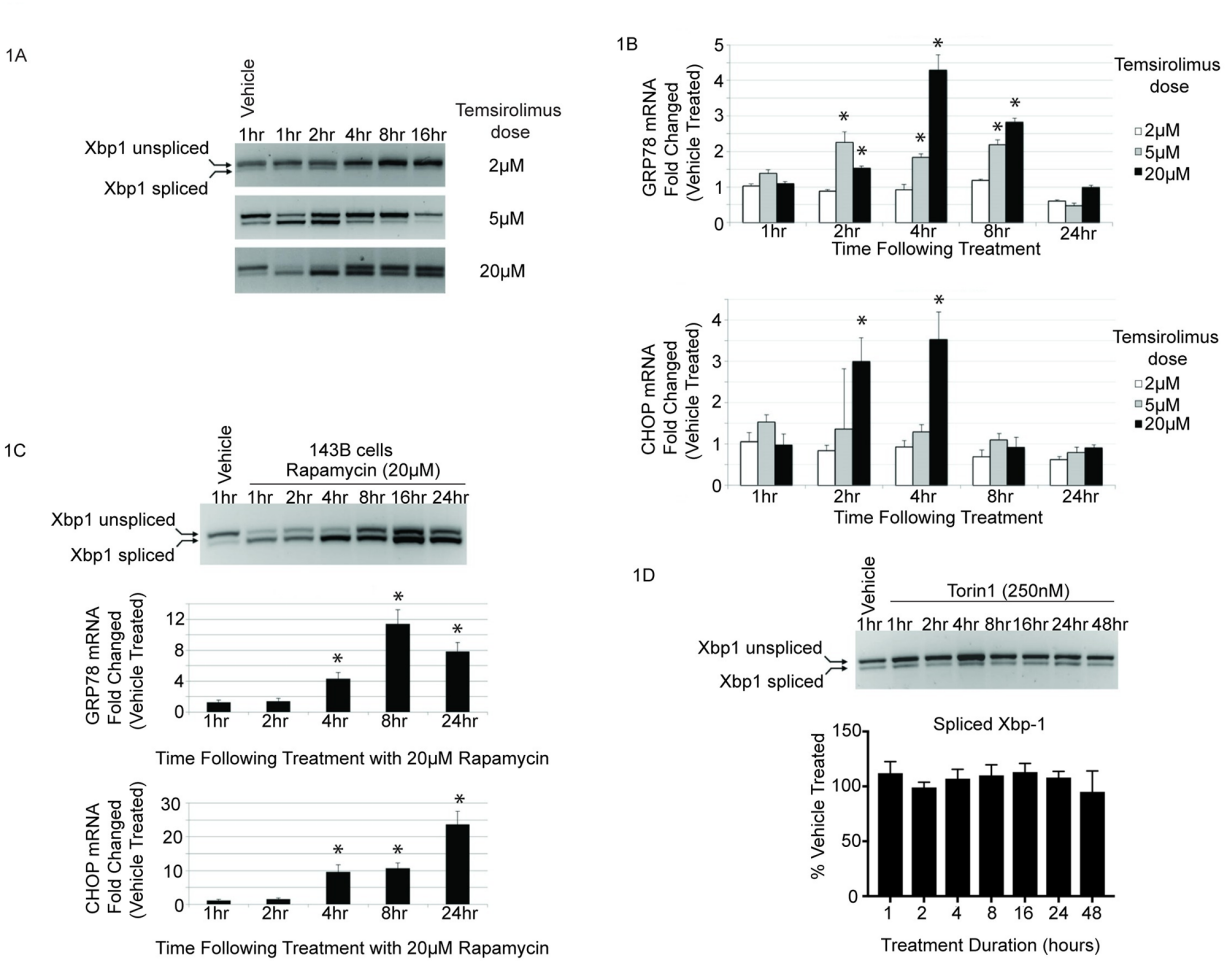
Rapamycin and temsirolimus induce Xbp-1 splicing and downstream target gene mRNA levels. **(A)** Human osteosarcoma cells (HOS-MNNG) were treated for the indicated times with 2, 5 or 20μM temsirolimus. RT-PCR was conducted using a primer pair flanking the unconventional splice site of the Xbp-1 mRNA. (**B**) Quantitative RT-PCR was conducted using primer pairs specific for the Xbp-1 target genes, Grp78 and CHOP. Expression values for each transcript were normalized to 18S and vehicle treated control. Experiments were carried out on three independent biological replicates, assayed in triplicate. One-way ANOVA was performed for statistical analysis and asterisk denotes *p* = <0.05. (**C**) 143B human osteosarcoma cells were treated with vehicle or 20μM rapamycin and analyzed for Xbp1 splicing (upper panel) and downstream target gene activation of BiP/Grp78 and CHOP at the indicated time points as in (A and B). (**D**) Cells were treated for the indicated times with either 20μM temsirolimus or an ATP competitive inhibitor of mTOR. Total RNA was harvested and Xbp-1 splicing was analyzed by RT-PCR as in (A).

As shown in Fig 1A, temsirolimus induced Xbp-1 splicing in a dose dependent manner in human osteosarcoma cells. Splicing occurred as early as 1hr following treatment and was transient. The ratio of spliced to unspliced Xbp-1 mRNA returned to basal levels approximately 4hrs following 5μM drug exposure and 24hrs after 20μM exposure. As stated previously, these exposures of rapamycin have been safely achieved in patients [20, 25–29]. The steady-state level of unspliced Xbp-1 mRNA increased just prior to the first detectable cleavage, consistent with previous reports demonstrating increased Xbp-1 message stability contributes to efficient IRE1 mediated splicing [4, 5]. Similar results were seen in other osteosarcoma and rhabdomyosarcoma cells, as well as human breast cancer cells, demonstrating that Xbp-1 splicing in response to temsirolimus was not a cell type specific effect (S1 and S2 Figs).

Next, we determined the extent of Xbp-1 target gene transcription, following temsirolimus treatment, using quantitative RT-PCR. As shown in Fig 1B, BiP/Grp78 and CHOP mRNA levels were transiently increased following temsirolimus treatment and were only induced at time points following initiation of Xbp-1 splicing. These effects were not limited to cells treated with temsirolimus as the parent compound, rapamycin, induced similar changes in Xbp-1 splicing and concomitant increases in BiP/Grp78 and CHOP mRNA levels, as shown in Fig 1C. Taken together, these results define the low micromolar exposures of temsirolimus or rapamycin that induce Xbp-1 splicing as well as downstream BiP/Grp78 and CHOP gene expression, molecular changes consistent with UPR activation.

### mTOR kinase inhibition using an ATP competitive inhibitor has no effect on Xbp-1 splicing

The classic mechanism of action for rapamycin/rapalogs is through inhibition of mTOR kinase activity resulting from direct interaction with drug bound FKBP12 [19, 30]. Previous studies have shown that low doses of rapamycin/rapalogs inhibit the mTORC1 arm of mTOR signaling, but over longer incubation times or at higher doses mTORC2 inhibition also occurs [31–38]. In order to achieve simultaneous and potent mTORC1/2 inhibition, new small molecule inhibitors have recently been described [39–41]. These drugs act as direct, ATP competitive inhibitors of mTOR kinase activity, distinct from the macrolide rapamycin and rapalogs. It would reason that if UPR induction by rapamycin/rapalogs occurred as a result of mTORC1/2 inhibition then a more potent and direct mTOR inhibitor should also induce Xbp-1 splicing. In order to test this hypothesis, cells were treated with an ATP competitive inhibitor for up to 48hrs. Importantly, this dose has been shown to inhibit both mTORC1 and mTORC2 arms of mTOR signaling (S3 Fig) and [41]. Interestingly, as shown in Fig 1D, the ATP competitive inhibitor failed to induce Xbp-1 splicing at any time point suggesting that mTOR inhibition is not sufficient to induce UPR. Similar results were seen with a second, structurally distinct, ATP competitive mTOR inhibitor (S3 Fig).

### Micromolar exposures of temsirolimus result in inhibition of total protein synthesis and increased eIF2 alpha phosphorylation

**Fig 2:**
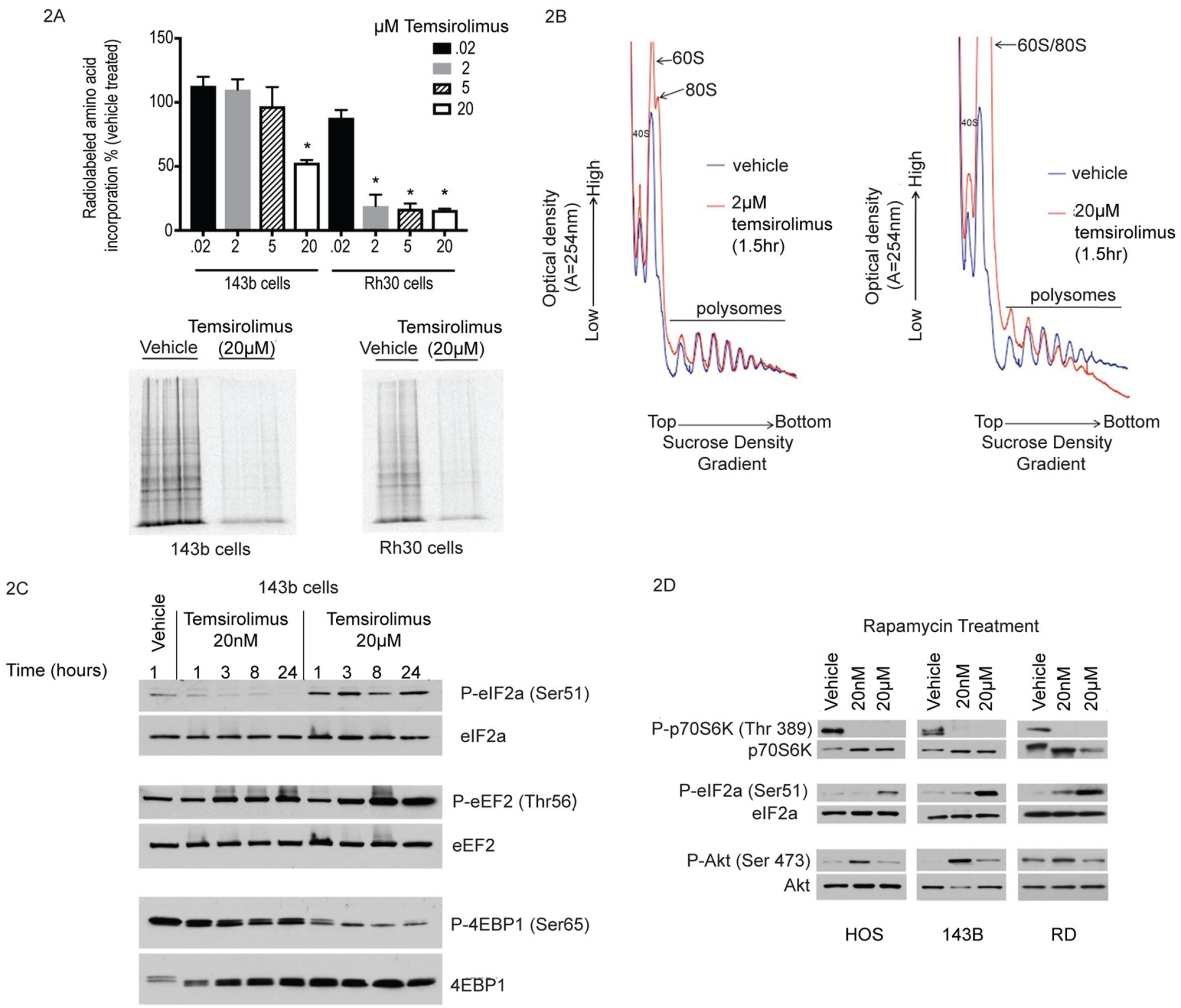
Identification of temsirolimus exposures required to inhibit total protein synthesis. (**A**) Cells were pulse labeled with ^35^S-methionine/cysteine and analyzed by scintillation counting for incorporated radioactivity normalized to vehicle treated control (top panel) or SDS-PAGE (lower panel). Experiments were performed in duplicate or triplicate and mean values shown +/- standard deviation. One-way ANOVA was performed for statistical analysis and asterisk denotes *p* = <0.05 for treatment condition compared to vehicle treated control. (**B**) Human rhabdomyosarcoma cells (Rh30) were treated with 2μM or 20μM temsirolimus for 1.5hrs. Cell lysates were then layered onto 15–45% linear sucrose gradients for polysome analysis. Samples were fractionated and UV absorbance was measured in real-time. The location of 40S, 60S ribosomal subunits as well as 80S ribosome monomers and larger molecular weight polysomes are indicated. (**C**) Human 143B osteosarcoma cells were treated with vehicle, 20nM or 20μM temsirolimus for the indicated times. Protein lysates were analyzed by Western blot for levels of phospho and total protein for eIF2 alpha, eEF2 and 4EBP1. (**D**) The same as in (A) using human osteosarcoma cells (HOS and 143B) as well as human rhabdomyosarcoma cells (RD) treated with vehicle, 20nM or 20μM rapamycin for 1hr. Three independent experiments were performed for each treatment condition.

Next, we sought to determine the phenotypic effects associated with UPR activation in response to temsirolimus. Another hallmark of UPR induction is inhibition of total protein synthesis [1, 42, 43]. However, the current paradigm is that rapamycin and rapalogs inhibit translation only of a specific subset of mRNA (5’ TOP mRNA) without effecting total protein synthesis [44, 45]. Therefore, we wanted to determine the extent to which micromolar exposures of temsirolimus, which induce Xbp-1 cleavage, also, inhibit total protein synthesis. In order to achieve this goal, we first conducted metabolic radiolabeling studies. As shown in Fig 2A, the amount of radiolabeled amino acids incorporated into newly synthesized protein was decreased in both osteosarcoma and rhabdomyosarcoma cells and only at micromolar doses.

Next, in order to further characterize effects on protein synthesis, we conducted polysome analysis by sucrose density gradient centrifugation. If total protein synthesis is inhibited, there should be a decrease in peak height for the heaviest polysomes (greatest number of ribosomes/mRNA) with a corresponding increase in 80S monosomes and smaller polysomes [46]. As shown in Fig 2B, vehicle treated cells exhibit well defined peaks corresponding to individual ribosomal subunits as well as 80S monomers and higher multimer polysomes which represent active translation. Following treatment with temsirolimus, the polysome peak area is decreased relative to vehicle treated samples and there is a dramatic increase in 80S monomers consistent with global inhibition of protein synthesis. These results are consistent with the metabolic radiolabeling data (Fig 2A) with a slight difference in the exposure of temsirolimus required to demonstrate translation inhibition in Rh30 cells depending on the assay used. In either case, a micromolar threshold level is required to inhibit protein synthesis.

In order to determine the step(s) at which translation was inhibited, the phosphorylation state of eIF2 alpha and eEF2 were analyzed. Under conditions which promote UPR activation, phosphorylation of both eIF2 alpha and eEF2 increase predicting inhibition of translation initiation and elongation, respectively [42, 43, 47, 48]. As shown in Fig 2C, phosphorylation of eIF2 alpha (Ser 51) increased as early as 1hr following treatment with 20μM, but not 20nM, temsirolimus. The phosphorylation of eIF2 alpha persisted through 24hrs and is consistent with long-term inhibition of translation initiation. This data is also supported by decreased polysome profiles observed through at least 12hrs which was the longest treatment duration analyzed by sucrose density gradient centrifugation (S4 Fig). In contrast, eEF2 phosphorylation was increased at either dose, although to a greater extent in 20μM temsirolimus treated samples. A similar increase in eIF2 alpha (Ser 51) phosphorylation was also observed in multiple human osteosarcoma and rhabdomyosarcoma cell lines treated with 20μM, but not 20nM rapamycin (Fig 2D). Taken together, our results are consistent with UPR associated inhibition of total protein synthesis. This seems to occur primarily at the level of initiation through sustained inactivation of eIF2 alpha, although we cannot rule out the combined effects of hyperphosphorylated eEF2 and/or altered activity of 4EBP1 and p70S6 kinase.

4EBP1 can undergo a hierarchical series of phosphorylation events which is thought to result in disruption of eIF4E binding [49]. This results in increased cap binding by eIF4E and increased protein synthesis. On the other hand, hypo-phosphorylation of 4EBP1 is thought to inhibit binding eIF4E to eIF4G, blocking pre-initiation complex formation and inhibiting protein synthesis. We specifically chose to examine 4EBP1 phosphorylation at serine 65, because it has been shown to be mTOR dependent and is the final phosphorylation event in the series of Thr37/46 followed by Thr65 and ultimately serine 65.

Ser65 phosphorylation of 4EBP1 was minimally reduced at 20nM, although phosphorylation of other sites in 4EBP1 (such as Thr37/46) is known to be eliminated at this dose. However, Ser65 phosphorylation is substantially reduced at 20μM, although the mechanism and significance of this finding is unclear. For example, a previous study demonstrated that complete inhibition of mTORC1/2 using the ATP competitive inhibitor Torin1 also resulted in a dramatic decrease in 4EBP1 Ser65 phosphorylation [41]. Therefore, this event appears to be mTOR dependent. This same study showed that Torin1 did not affect the phosphorylation of eIF2a (Ser51) and we did not detect increased Xbp1 splicing (Fig 1D) [41]. These distinctions provide additional evidence that biomarkers of the unfolded protein response, Xbp1 splicing and eIF2a (Ser51) phosphorylation, induced by micromolar exposures of rapamycin and rapalogs are involved in a second mechanism of action that is mTOR independent. These data challenge a long held view that rapamycin and rapalogs have limited effects on global translation regulation and provide the mechanistic basis for this to occur as a result of UPR induction.

### Biphasic growth inhibition characterized by FK-506 sensitive and insensitive mechanisms

**Fig 3:**
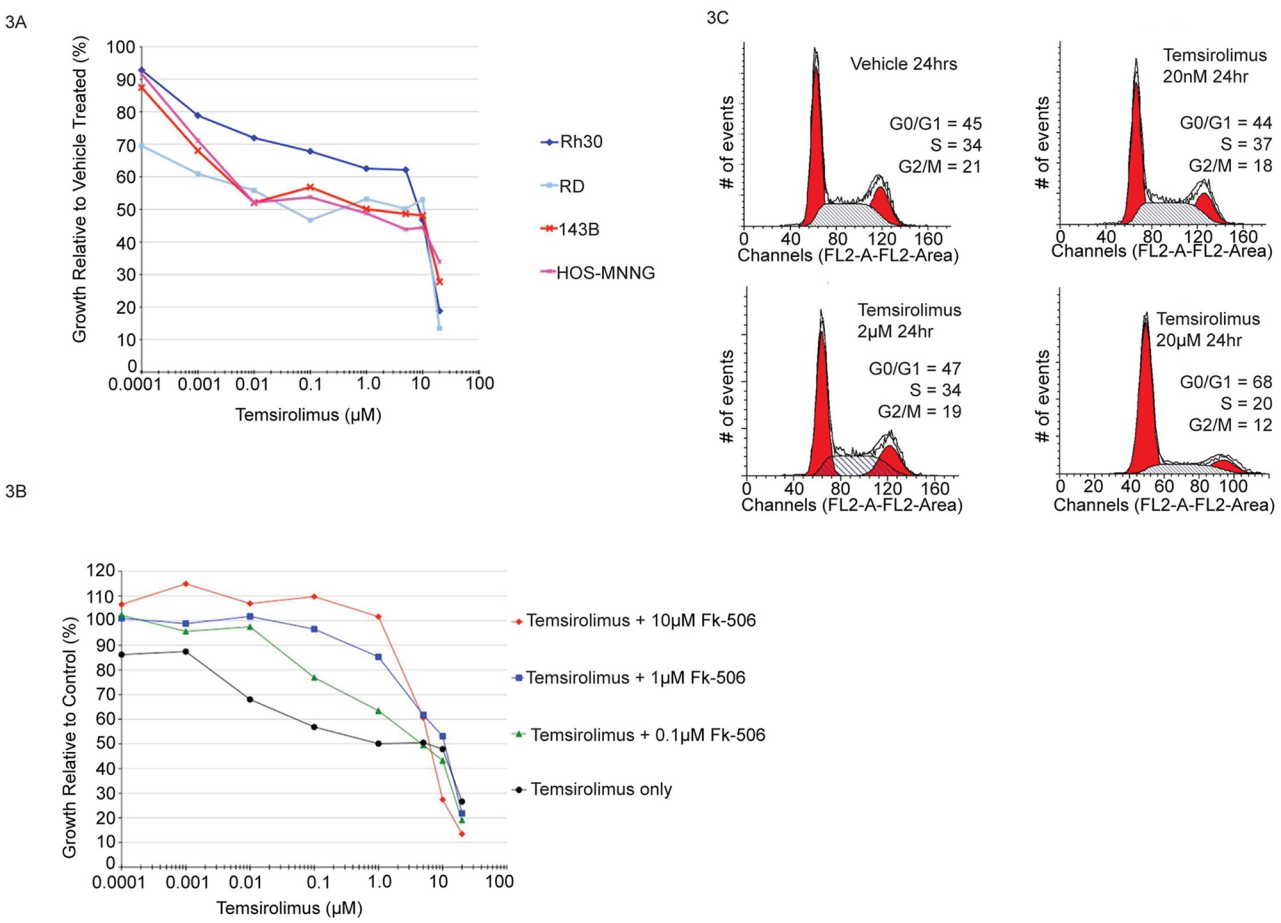
Pharmacologically relevant micromolar doses of temsirolimus provide maximal tumor cell growth inhibition. (**A**) Human rhabdomyosarcoma (Rh30, RD) and osteosarcoma (143B, HOS-MNNG) cells were treated for 48hrs with the indicated doses of temsirolimus followed by staining with sulforhodamine B. Growth is represented as percentage of vehicle treated control. Each cell line was assayed at least three times and mean values for each data point are shown. (**B**) Cell cycle analysis of asynchronous cells following treatment with temsirolimus at different doses. Human osteosarcoma cells were treated for the indicated times with vehicle, 20nM, 2μM or 20μM temsirolimus followed by FACS analysis of propidium iodide stained cells to determine the distribution of cells within each phase of the cell cycle. (**C**) Treatment with FK-506 rescues low dose growth inhibitory effects of temsirolimus, but not high dose effects. Human osteosarcoma cells were treated with the indicated doses of temsirolimus alone or in combination with FK-506. Both drugs bind the intracellular protein FKBP12, but only temsirolimus/FKBP12 complex can bind to mTOR. Treatment was for 48hrs. Cell growth is expressed relative to FK-506 alone and assayed by staining with sulforhodomine B. Each set of conditions were analyzed in three separate experiments and mean values are shown.

Next, we sought to determine the effect of temsirolimus on osteosarcoma and rhabdomyosarcoma cell growth. Temsirolimus was chosen for the analysis because it is currently being evaluated in clinical trials for solid tumors including sarcoma. Drug concentrations tested spanned the range shown to be clinically achievable in patients (1nM-20μM) [20, 25–29]. As shown in Fig 3A, single dose temsirolimus treatment induced biphasic growth inhibition in each cell line tested. The GI50 (drug concentration required to achieve 50% growth inhibition compared to vehicle treated) for each OS and RMS cell line were within the mean range of relative drug sensitivity when compared to GI50 values for the NCI-60 cell line panel (S5 Fig). These results demonstrate that the osteosarcoma and rhabdomyosarcoma cells used for these studies are not uniquely sensitive or resistant compared to other tumor cell types.

In contrast to continuous 48hr drug exposures, we wanted to determine the extent to which shorter duration exposures were capable of inhibiting tumor cell growth. Cells were incubated in the presence or absence of 20μM temsirolimus for 4hrs (or 24hrs) after which growth medium was removed. Cells were then washed repeatedly and fresh medium without drug was added for the final 44hrs or 24hrs (respectively) of incubation prior to assaying cell growth. Cells were also incubated for the full 48hrs exposed to drug as well. A single 4hr exposure was sufficient to result in >50% growth inhibition measured at 48hrs (S6 Fig). These data suggest that short term, high dose temsirolimus treatment, at drug concentrations achievable in patients, is capable of effectively inhibiting tumor cell growth and that chronic dosing may not provide additional benefit.

In order to characterize the mechanism(s) leading to growth inhibition following temsirolimus treatment, we conducted cell cycle analysis. As shown in Fig 3B, there were no changes in cell cycle distribution for unsynchronized cells treated with 20nM or 2μM temsirolimus. However, 20μM treatment resulted in altered cell cycle progression with an increase in the proportion of G1 phase cells. These results are consistent with a cytostatic effect and support previous studies describing UPR induced G1 arrest as a consequence of translation inhibition [47]. It is likely that the modest low dose growth inhibitory effect observed over a broad range of temsirolimus concentrations (Fig 3A) results from a decrease in the rate of cell cycle progression whereas the high dose effect is characterized by cells arresting and therefore, accumulating in G1 phase.

In order to further determine whether the low dose and high dose growth inhibitory effects were mediated by the same, prototypical mechanism of action, we conducted drug competition experiments using the related compound FK-506 (tacrolimus). The K(d) for rapamycin/FKBP12 and FK-506/FKBP12 complexes is nearly identical (~0.2nM) [50]. However, FK-506/FKBP12 cannot bind to the FRB domain of mTOR and therefore, does not inhibit mTOR kinase activity. On the contrary, competition with FK-506 has been shown to effectively rescue the inhibitory effects of rapamycin/temsirolimus on mTOR protein function and associated cell phenotypes [20]. Consistent with these previous studies, in human OS and RMS cells, FK-506 rescued the low dose growth inhibitory effects of temsirolimus when present in equimolar concentrations up to 1μM (Fig 3C). However, no antagonism was observed at higher micromolar exposures (10μM) of each drug further supporting an alternative, non-canonical mechanism of action.

A previous study suggested that temsirolimus can directly interact with mTOR, independent of FKBP12, and that this may account for the observed high dose drug effects and represent a new mechanism of action [20]. While we cannot rule out this possibility, previous work and our current data question this assertion. These previous results were only shown using purified recombinant mTOR/FRB and, to the best of our knowledge, such a direct interaction between mTOR/FRB and rapamycin/temsirolimus has never been shown in intact cells. In addition, FKBP12 is expressed in most cell types and the affinity for rapamycin is ~2,000X greater than the affinity of mTOR/FRB for rapamycin [30]. Therefore, while *in vitro* binding experiments, using recombinant protein may demonstrate rapamycin or temsirolimus/mTOR interaction is possible, it is not likely to be relevant under physiological conditions. Furthermore, unrelated and potent mTORC1/mTORC2 inhibitors (Torin1 and AZD-8055), effectively inhibit mTORC1/2 activity without inducing Xbp1 splicing, a defining feature of the unfolded protein response (Fig 1D and S3 Fig). In addition, a previous study demonstrated that Torin1 failed to induce eIF2a (Ser51) phosphorylation [41]. Taken together, our data argue against a direct role for mTOR/FKBP12 in mediating the high dose, UPR inducing effects of rapamycin and temsirolimus in tumor cells.

### Decreased 28S ribosomal RNA solvent accessibility in cells treated with temsirolimus

Rapamycin is a naturally occurring macrocyclic polyketide synthesized by *Streptomyces hygroscopicus* [51]. It is structurally related to the general class of antibiotics known to inhibit prokaryotic protein synthesis through direct interaction with the 50S, large ribosomal subunit. In support of the potential for direct interaction between rapamycin and the ribosome, a previous study determined the crystal structure of rapamycin bound to the large ribosomal subunit of the eubacterium *Deinococcus radiodurans* [52, 53]. Chemical mapping determined that the interaction occurs through several contacts between rapamycin and 23S rRNA along the nascent polypeptide exit tunnel.

**Fig 4:**
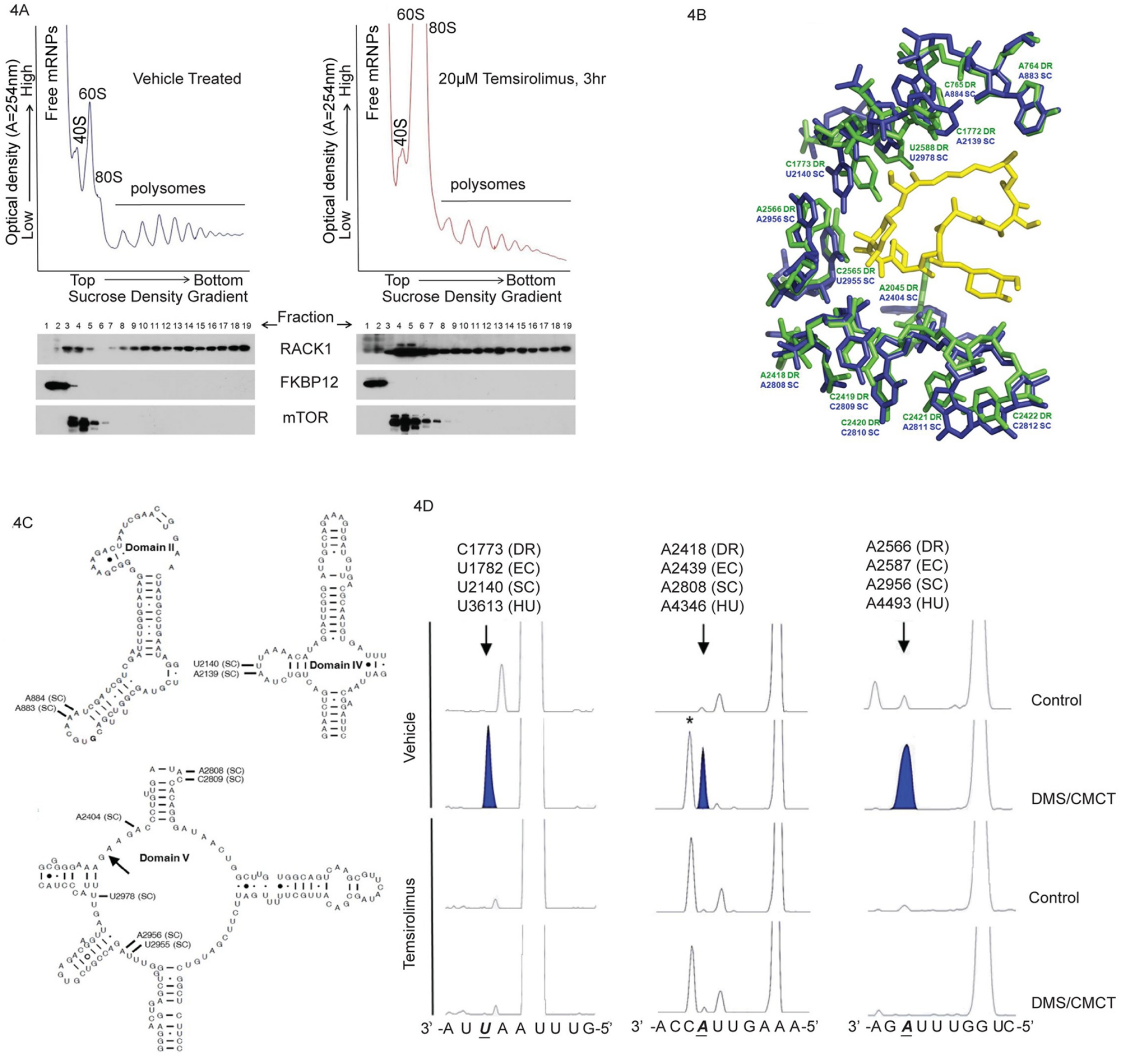
Decreased solvent accessibility of 28S rRNA in ribosomes isolated from cells treated with temsirolimus. (**A**) Polysome profiles (upper panel) and Western blot analysis of mTOR and FKBP12 protein levels (middle panel) in fractions following sucrose density gradient fractionation. Temsirolimus levels were quantified by HPLC-MS-MS analysis from pooled fractions as indicated in the text. **(B**) Alignment of X-ray crystal structures from *D*. *radiodurans* complexed with rapamycin (PDB 1Z58) and *S*. *cerevisiae* (PDB 3U5D) [52, 54]. (**C**) Secondary structure model for *S*. *cerevisiae* large subunit domains II, IV and V, adapted from the Comparative RNA Website [55]. Arrow denotes location of the entrance to the peptide exit tunnel and the location of nucleotides in (B) are labeled. (**D**) Protection of 28S rRNA from chemical modification in temsirolimus treated samples. Primer extension analysis of human 28S rRNA from ribosomes isolated from temsirolimus treated or vehicle treated human rhabdomyosarcoma cells. Purified ribosomes were treated with DMS (modifies accessible adenosine and cytosines), CMCT (modifies accessible uracil and guanines) or mock treated. RNA was then purified, quantitated and equal amounts were used as the template for fluorescent primer extension analysis. Capillary electrophoresis was used to separate and resolve products under conditions allowing for single nucleotide resolution along with a DNA size standard. Arrows and shaded peaks denote the position of a DMS or CMCT modifiable ribonucleotide which is protected from modification in temsirolimus treated samples. An asterisk (*) denotes a chemical independent stop in some samples. The human nucleotide sequence appears below the electropherogram with *D*. *radiodurans* (DR), *E*. *coli* (EC), *S*. *cerevisiae* (SC) and *H*. *sapiens* (HU) numbering to denote the position of interest. Nucleotide positions were adjusted (+1) to account for the fact that DMS and CMCT modification results in a stop one nucleotide before the modified base.

Based on the evolutionarily conserved structure/function relationship between prokaryotic and eukaryotic ribosomes, we hypothesized that rapamycin/rapalogs may interact with human ribosomes. In order to test this hypothesis, we first quantified temsirolimus levels from treated cells following sucrose density gradient centrifugation. Temsirolimus was detected in ribosome fractions at all time points analyzed (1.5, 3 and 12hrs) following treatment (S1 Table). The amount of temsirolimus in heavy polysome fractions decreased over time and correlated with a decrease in the amount of ribosomes in each of these fractions (Figs 2A and 4A and S4 Fig). Since temsirolimus has only been shown to bind FKBP12 in cells, we analyzed samples for the presence of FKBP12 protein, as well as mTOR, in order to determine if this would account for the presence of drug in each fraction. As shown in Fig 4A, FKBP12 was detected primarily in free mRNP fractions whereas mTOR was detected in fractions containing 40S, 60S and 80S particles. Neither protein was found in polysome fractions in which temsirolimus was detected. In addition, we detected little overlap in FKBP12 and mTOR containing fractions suggesting that temsirolimus binding and ternary complex formation with each protein results in exclusion from mRNPs, ribosomal subunits and intact ribosomes.

Next, we used published, high resolution structures for *S*. *cerevisiae* ribosomes to align with the structure of rapamycin bound *D*. *radiodurans* large ribosomal subunit [52, 54]. As shown in Fig 4B, alignment of the structures revealed that rapamycin is within hydrogen bonding distance to U2955 SC (C2565 DR), U2140 SC (C1773 DR), C2809 SC (C2419 DR) and A2404 SC (A2045 DR). In addition, several hydrophobic interactions are possible involving nucleotides from domains II, IV and V of the large ribosomal subunit (Fig 4C). These residues are completely conserved between *S*. *cerevisiae* and humans, although we cannot rule out the possibility that structural differences exist. However, this information is supported by chemical probing data as shown in Fig 4D. Ribosome fractions were treated with dimethyl sulfate (DMS) or 1-cyclohexyl-3-(2-morpholinoethyl) carbodiimide metho-*p*-toluenesulfate (CMCT) which react with nucleotides not involved in Watson-Crick base pairing or drug interaction [56]. This RNA served as the template for subsequent primer extension analysis in order to map the position of modified and protected nucleotides. Human A4493 (A2956 SC, A2566 DR) and A4346 (A2808 SC, A2418 DR) were susceptible to DMS modification in vehicle treated samples yet protected in samples from temsirolimus treated cells. Similarly, temsirolimus treatment also resulted in protection of human U3613 (U2140 SC, C1773 DR) from CMCT modification.

One key difference between the structures is the relative conformation of nucleotide A2045 DR (A2404 SC). It should be noted that this nucleotide has been shown to be highly flexible and to undergo conformational changes upon macrolide binding as well as interact with the nascent chain [57, 58]. As positioned in the *S*. *cerevisiae* structure, shown in Fig 4B, the ribose O2’ of A2404 SC is within hydrogen bonding distance to the O2’ of rapamycin. This is in contrast to the *D*. *radiodurans* structure where the O2’ is positioned further away due to rotation about the glycosidic linkage which then supports hydrophobic interaction between the base and rapamycin. Another difference between the structures is the position of C1773 DR (U2140 SC). This appears to be a function of rapamycin binding as the base is normally involved in tertiary interactions with C2565 DR (S7 Fig) [55]. Similar tertiary interactions are observed for U2140 SC and U2955 SC and the actual position and conformation of these nucleotides in the presence of rapamycin will require additional structural data from higher eukaryotes.

Although previous work, using ribosomes isolated from *D*. *radiodurans*, failed to show inhibition of protein synthesis in response to rapamycin, there is no functional equivalent of UPR in prokaryotes. Based on the structural data, it does not appear that the presence of rapamycin within the peptide exit tunnel would physically block the path by which nascent chains progress [52]. However, growing peptides must pass immediately adjacent to the rapamycin binding region as they approach the tunnel constriction formed by ribosomal proteins L4 and L22 (L17 in eukaryotes). This region of the tunnel has been shown to be involved in co-translational folding and stabilization of the nascent peptide [58, 59]. In addition, this region is important for sensing the presence of transmembrane (TM) segments in growing peptides and coupling this to structural rearrangements at the ER membrane, specifically opening and closing of the translocon pore [12]. Proper gating is required to maintain the integrity of the pore otherwise an unregulated efflux of calcium from the ER may occur, an event known to induce UPR [60]. In addition, proper gating of the translocon allows for multipass proteins to be properly inserted into the ER membrane [61]. Disruption of this process could result in these proteins being presented to the ER as misfolded peptides resulting in UPR induction. In order to distinguish between these possibilities, future studies will be needed.

Mitogenic signaling stimulates mTOR kinase activity resulting in increased protein synthesis [19, 31, 34]. However, cells treated with rapamycin/rapalogs have decreased mTOR kinase activity and decreased phosphorylation of 4E-BP1 and S6K1 [19, 31–36, 44]. This information provided the rationale for using these proteins as biomarkers for studies evaluating rapamycin/rapalogs in clinical trials. However, phosphorylation of these mTOR targets is inhibited at doses far below those typically needed for therapeutic effectiveness. Therefore, it appears that these classic, proximate targets of mTOR may be highly sensitive biomarkers of rapamycin exposure, but not of effective exposure or of an exposure predictive of therapeutic response.

It is clear that at least two distinct levels of effect can be attributed to rapamycin/rapalogs. Modest, cytostatic responses in tumor cells (mTORC1 dependent effects) generally occur over a broad range of rapamycin/rapalog doses typically in the picomolar to high nanomolar range along with dramatic decreases S6K1 phosphorylation. In addition, these conditions have been shown to promote hyper phosphorylation of Akt over time due, in part, to loss of feedback inhibition through IRS1 [22, 38, 62]. However, a second and distinct level of activity is seen at low micromolar doses and this is associated with maximal inhibition of tumor proliferation *in vitro* and in murine models [20].

The mechanism of action we describe for rapamycin and temsirolimus, may immediately inform their use in patients with cancer. Based on the results, it is clear the second mechanism of action for rapamycin and rapalogs is dependent on maximum concentration. In patients in clinical trials, this value is reflected as c_max_. Specific c_max_ values can range as high as 2–20μM depending on the clinical trial [20, 25–29]. The low anti-tumor efficacy of rapamycin and rapalogs in some clinical trials may be due to failure to induce the unfolded protein response. In addition, it is possible that patients in previous trials subjected to continuous dosing may have experienced dose-limiting toxicity. We believe our current work provides the rationale for examining intermittent dosing which may be better tolerated by patients. Evaluation of pharmacodynamic biomarkers such as eIF2a (Ser51) phosphorylation, as an indicator of protein synthesis inhibition, and Xbp1 splicing, as a measure of UPR induction, would be useful and may better correlate with a positive clinical response.

## Materials and methods

### Cells and chemicals

Human osteosarcoma (OS) cell lines (HOS-MNNG and 143B) human rhabdomyosarcoma (RMS) cell lines (RD, alveolar RMS and Rh30, embryonal RMS) and the human breast cancer cell line MCF7 were obtained from the American Type Culture Collection (ATCC). Cells were grown in DMEM supplemented with 10% fetal bovine serum, penicillin, streptomycin, L-glutamine and incubated at 37°C with 5% CO_2_ and 100% humidity. FK-506 (tacrolimus, LC Laboratories), CCI-779 (temsirolimus, LC Laboratories), rapamycin (sirolimus, LC Laboratories), Torin1 (kind gift from Dr. Nathanael S. Gray, Dana-Farber Cancer Institute and David M. Sabatini, Whitehead Institute/MIT/HHMI), thapsigargin (#T-9033, Sigma-Aldrich Corporation) and AZD-8055 (kind gift from Astra Zeneca Corporation).

### RNA isolation RT-PCR for Xbp-1 splicing

Cells were plated at a density of 1x10^5^ cells in a 60mm dish in complete media overnight followed by treatment with vehicle or drug for the indicated times. RNA was isolated from cells using Trizol LS^®^ (Invitrogen Corporation) reagent according to manufacturer’s instructions. RNA was subjected to an additional purification step using the RNeasy spin column clean up protocol supplied by the manufacturer (Qiagen Corporation). Samples were quantitated using a Nanodrop apparatus. A total of 100ng of RNA was reverse transcribed using random hexamers and Moloney murine leukemia virus reverse transcriptase (Promega Corporation). Reverse transcription reactions were incubated for 1hr at 37°C and halted by heating at 70°C for 10 min. 2μl of cDNA was used for PCR reactions using TITANIUM^™^ *Taq* DNA polymerase (Clontech) and the following primers: human Xbp1 forward primer 5’-GAGTAGCAGCTCAGACTGCCAGAG-3’ and human Xbp1 reverse primer 5’-CAGACTCTGAATCTGAAGAGTCAATAC-3’. Thermocycling conditions were 95°C for 3 min followed by 28 cycles of: 95°C for 40 sec, 61°C for 40 sec and 68°C for 40 sec. PCR products were visualized following electrophoresis on 3% NuSieve^®^ 3:1 agarose gels (Cambrex Bioscience) in 1X TBE stained with GelStar^®^ nucleic acid gel stain (Cambrex Bioscience).

### Western blot analysis

Cells were seeded at a density of 3x10^5^ cells in a 10 cm dish in complete media overnight followed by treatment with vehicle or drug for the indicated times. Cell lysates were prepared using a NP-40 lysis buffer (include recipe) including protease and phosphatase inhibitors (Roche). Protein concentrations were determined and equal amounts for each sample were analyzed by SDS-PAGE. Primary antibodies used for immunoblot analysis were: FKBP12 (#3635–100, Biovision), eIF2 alpha (#9722, Cell Signaling Technologies), phospho-eIF2 alpha (Ser51) (#3398, Cell Signaling Technologies), eEF2 (#2332, Cell Signaling Technologies), phospho-eEF2 (Thr56) (#2331, Cell Signaling Technologies), Akt (#9272, Cell Signaling Technologies), phospho-Akt (Ser473) (#9271, Cell Signaling Technologies), phospho-Akt (Thr450) (#9267, Cell Signaling Technologies), mTOR (#2983, Cell Signaling Technologies), RACK1 (#610178, BD Biosciences), p70 S6 kinase (#2708, Cell Signaling Technologies), 4E-BP1 (#9644, Cell Signaling Technologies), phospho-4E-BP1 (Ser 65) (#9451, Cell Signaling Technologies).

### Sulforhodamine B (SRB) cell proliferation assay

Cells were plated in a 96-well plate at a density of 5,000–10,000 cells per well in 100μl of complete media and incubated overnight. Cells were then treated with vehicle or drug(s) as indicated and processed after 48hrs as previously described unless otherwise stated [63].

### Quantitative RT-PCR (QRT-PCR)

Cells were plated and RNA was isolated and reverse transcribed as mentioned above. An aliquot of diluted cDNA was used as template for quantitative PCR using the iQ SYBR Green Supermix and assayed using an iQ5 real time thermocycler (Bio-Rad Laboratories, Inc.). The following primers were used at a final concentration of 100nM for gene specific amplification: CHOP/GADD-153 forward primer 5’-TCTGATTGACCGAATGGTGA-3’, CHOP/GADD-153 reverse primer 5’-TCTGGGAAAGGTGGGTAGTG-3’, BiP/Grp78 forward primer 5’-TTTCACAGTGCCCAAGAGTG -3’, BiP/Grp78 reverse primer 5’-TGATCACTCACTCCCCATCA -3’ and 18S ribosomal RNA forward primer 5’-GTAACCCGTTGAACCCCATT-3’, 18S ribosomal RNA reverse primer 5’CCATCCAATCGGTAGTAGCG-3’.

### Polysome analysis by sucrose density gradient fractionation

Following vehicle or drug treatment, cells were incubated with cycloheximide (200μg/ml) for 10 min at 37°C. Samples were then placed on ice and washed twice with ice-cold PBS also containing cycloheximide. Cells were then lysed in polysome lysis buffer (250mM KCL, 10mM Tris-HCl, pH 7.4, 25mM MgCl_2_, 0.5% NP-40, 0.5% sodium deoxycholate, 1μ/μl Rnasin, 200μg/ml cycloheximide, EDTA-free protease inhibitors (Roche), phosphatase inhibitors (HALT phosphatase inhibitor cocktail, Pierce Chemicals) and nuclease-free water and incubated on ice for 15 min followed by centrifugation at 4°C for 10 min at 12,000 x *g*. Supernatant was then layered on to 15–45% (w/v) sucrose gradients containing 150mM KCl, 10mM Tris-HCL, pH 7.4, 25mM MgCl_2_, 200μg/ml cycloheximide and nuclease-free water. Gradients were formed using a Biocomp gradient former. Samples were centrifuged at 180,000 x *g* for 2.5 hrs at 4°C. An ISCO Model 640 fractionator equipped with a UV-6 absorbance monitor, 10mm path length flow-cell, was used to measure the optical density of the gradients at 254nm in real-time. A Dataq model DU-158 analog-to-digital converter was used for data acquisition using Windaq software (Dataq Corporation). Raw OD data was exported to Excel and graphed as absorbance (*A* = 254nm), y-axis, over time (seconds), x-axis. Area under the curve for the indicated regions was calculated using Graphpad software (Graphpad Prism).

### Isolation of protein from polysome fractions

Polysome fractions were treated with methanol/chloroform as previously described in order to precipitate protein for immunoblot analysis [64].

### Metabolic radiolabeling

Cells were pre-incubated with vehicle or drug for 2hrs, then pulse labeled for 30 min using ^35^S-methionine/cysteine. Cells were subsequently lysed and protein was precipitated with trichloroacetic acid (TCA). Incorporation of radiolabeled amino acids into newly synthesized protein was determined by scintillation counting. Samples were also analyzed by SDS-PAGE and radioactivity quantified using a Phosphorimager.

### Quantitation of temsirolimus and rapamycin

For the analysis of whole cell lysates and polysomal fractions, the following volumes were used: 150 μl of whole cell lysate, 500 μl of fractions 1–3, 1 ml for fractions 6–10, and 2 1.5 ml aliquots (combined organic prior to dry down) for fractions 11–20. To each sample, 500 μl of 100 mM ammonium acetate, pH 4 was added with brief mixing, followed by 4 ml of diethyl ether and extraction by vortexing for 8 min. Organic and aqueous layers were separated by centrifugation (8 min, 2,400 x *g*) and the organic layer removed, evaporated by vacuum centrifugation at room temperature and reconstituted in 100 μl of beginning mobile phase (60% methanol: 40% 10 mM ammonium acetate, pH 4.7) for LC/MS-MS analysis.

Positive ion electrospray ionization (ESI) mass spectra were obtained with an AB Sciex 3200 QTRAP^™^ triple quadrupole mass spectrometer (Foster City, CA) with a turbo V^™^ ion source interfaced to a Shimadzu HPLC system. Samples were chromatographed with a Waters Sunfire C18, 2.5um, 50mm×4.6 mm column (Milford, MA). The LC was a gradient elution utilizing 100% methanol as the organic phase and 10 mM ammonium acetate, pH 4.7 as the aqueous as follows: 60% methanol for 0.75 min, linearly ramp to 99% methanol at 1.25 min, hold at 99% for 1.75 min, return to 60% methanol over 30 sec and equilibrate column for 30 sec at 60% methanol. The flow rate was 0.9 ml/min and sample injection volume of 60 μl. The analysis time was 4 min. The mass spectrometer settings were: temperature, 550°C; spray needle, 5500V; curtain gas, 10; collision gas, N_2_ (CAD), 2; ion source gas 1 and 2; both 60. The compound dependent settings for temsirolimus and rapamycin were as follows, respectively: declustering potential, 107 and 36.61; excitation potential, 11 and 6.3; collision cell entrance potential, 77 and 61.89; collision energy, 74 and 33; and collision cell exit potential, 4 and 7.61. Samples were quantified in the MRM mode by monitoring the transition *m/z* 1052.5 to 461.2 for temsirolimus and *m/z* 931.51 to 864.4 for rapamycin. Dwell time for each transition was 250 ms. Quantitation of temsirolimus and rapamycin were based on standard curves in water using 1/*x*^2^ weighting for each analyte.

### Chemical probing of rRNA and fluorescent primer extension analysis

Ribosomes from temsirolimus treated cells were isolated as described previously [65]. Samples were suspended in a buffer containing 30mM Hepes-KOH (pH 7.6), 70mM KCl, 0.25M sucrose, 5mM MgCl_2_ and 5mM 2-mercaptoethanol. Ribosomes (100pmol) in final volumes of 50μl were modified for 5 min at 37°C in the presence of 100mM dimethyl sulfate (DMS, Sigma-Aldrich) or for 15 min at 37°C in the presence of 1-cyclohexyl-3-(2-morpholinoethyl) carbodiimide metho-*p*-toluenesulfate (CMCT, Sigma-Aldrich). Control reactions were treated identically, but without the modifying agent. Reactions were quenched and RNA was precipitated with 2.5 volumes of 100% ethanol and 0.1 volumes of 3M NaCH_3_COO (pH 5.2) followed by centrifugation. Pellets were dissolved in 0.1M Tris-HCl (pH 7.6) and 0.5% (w/v) SDS followed by phenol/chloroform extraction of RNA and precipitation as above. Primer extension analysis was performed using several DNA primers designed to complement 28S rRNA regions located in the vicinity of the crystallographically determined macrolide binding pocket and rapamycin binding region: 28S domain IV 5’-/6-FAM/gcgggccttcgcgatgc-3’, domain V 5’-/6-FAM/ccgccacaagccagttatcc-3’, domain V 5’-/6-FAM/caacaacacatcatcagtagg-3’. cDNA synthesis was conducted using Superscript III reverse transcriptase (Invitrogen, Carlsbad, CA) according to manufacturers’ instructions with the following modifications. RNA was diluted in 9μl of annealing buffer (50mM Tris-Cl, pH 8.3, 60mM NaCl, 10mM DTT) and added to 1μl of fluorophore-labeled primer stock solution (1μM) to each tube. Samples were heated to 85°C for 1 min, followed by slow cooling to 25°C for primer annealing, then 9μl of reverse transcription mix was added (4μl of 5X RT buffer supplied with Superscript III, 1ul of 0.1M DTT, 2μl of RNase Inhibitor, 2μl of 10mM dTNP mix) in each tube. Samples were incubated at 55°C for 5 min then 200U of reverse transcriptase was added. The final reaction volume is 20μl which was then incubated at 55°C for 120 min. Upon completion of primer extension, RNA was degraded by adding 2μl of 2N NaOH and incubating at 95°C for 3 min. Reactions were neutralized by adding 2μl of 2N HCl followed by 3μl of 3M Na-acetate to facilitate cDNA precipitation by adding 80μl of 100% ethanol. Samples were centrifuged at 14,000rpm for 30 min which was then air-dried and resuspended in 11μl of Hi-Di formamide (Applied Biosystems) and 0.5μl of Genescan 500 ROX DNA size standard (Applied Biosystems). cDNAs were separated by capillary electrophoresis in an Applied Biosystems 3530 genetic analyzer. Results were analyzed using Peak Scanner software (Applied Biosystems).

### Analysis of X-ray crystal structure data

Structural data for *D*. *radiodurans* and *S*. *cerevisiae* large ribosomal subunits were accessed from the Protein Data Bank (http://www.rcsb.org/pdb/home/home.do). Data were analyzed in Pymol v1.5 (www.pymol.org) including alignment of structures. Bond distances and hydrophobic contacts were determined in Pymol and Ligand Explorer. Figures were made using Pymol.

## Supporting information

S1 Fig Dose dependent splicing of Xbp-1 mRNA in response to temsirolimus in human Rh30 rhabdomyosarcoma (RMS) cells. Cells were treated for the indicated times and doses of temsirolimus. Total RNA was harvested and RT-PCR was conducted using a primer pair flanking the unconventional splice site of the Xbp-1 mRNA. PCR amplicons were separated on an agarose gel stained with Gelstar reagent and visualized on a UV transilluminator equipped with a CCD camera.
(TIF)

S2 Fig Rapamycin induces splicing of Xbp-1 mRNA in human breast cancer cells (MCF7). Cells were treated for the indicated times and dose of rapamycin (sirolimus). Total RNA was harvested and RT-PCR was conducted using a primer pair flanking the unconventional splice site of the Xbp-1 mRNA. PCR amplicons were separated on an agarose gel stained with Gelstar reagent and visualized on a UV transilluminator equipped with a CCD camera.
(TIF)

S3 Fig Inhibition of mTOR dependent p70S6K and Akt phosphorylation by the mTOR small molecule inhibitors AZD-8055 or Torin1. Human 143B osteosarcoma cells were treated for the indicated times and doses of AZD-8055 or Torin1. Total protein was harvested and analyzed by Western blot (upper panels) or total RNA analyzed by RT-PCR (bottom panel) as described in Materials and Methods.
(TIF)

S4 Fig Inhibition of total protein synthesis by temsirolimus 12hrs post treatment. Human Rh30 rhabdomyosarcoma cells were treated for 12hrs with the indicated dose of temsirolimus. Post-mitochondrial supernatant was layered on 15–45% sucrose density gradients and fractionated as described in Materials and Methods. The location of free mRNPs, 40S and 60S ribosomal subunits, 80S monosomes and polysomes are noted. The optical density (*A* = 254nm) was monitored in real-time and plotted along the y-axis. Gradient depth is plotted along the x-axis.
(TIF)

S5 Fig Relative growth inhibitory (50%) concentrations of temsirolimus for the cell lines used in our current study compared to the NCI-60 cell line panel. Data for the NCI-60 cell lines was obtained from the Developmental Therapeutics Program (DTP) at the National Cancer Institute. Cell lines used in our current study (143B, Rh30, RD, MCF7) were assayed using the same assay and methodology as published for the NCI-60 panel [63]. Values for all cell lines were mean centered and plotted in order to demonstrate that the cells used for this study were not uniquely sensitive or resistant compared to other tumor cell lines.
(TIF)

S6 Fig Short-term micromolar exposures to temsirolimus are capable of causing long-term growth inhibition. Human 143B OS cells were treated for the indicated durations with 20μM temsirolimus and assayed for growth, compared to vehicle treated, at 48hrs. For example, cells were exposed to 20μM temsirolimus for 4hrs, extensively washed to remove drug and then refed with growth medium in the absence of drug for another 44hrs. Samples were then compared to vehicle treated using the sulforhodamine B assay as described in Materials and Methods.
(TIF)

S7 Fig Comparison of X-ray crystal structures for *D*. *radiodurans* and *S*. *cerevisiae* around the putative rapamycin binding region. The left panel is the result of alignment between *D*. *radiodurans* (dark blue) in the native state without rapamycin bound and *S*. *cerevisiae* (cyan) [54, 66]. The right panel is the result of an alignment between rapamycin bound (green) and unbound (blue) *D*. *radiodurans* X-ray crystal structures [52, 66]. All alignments, root-mean squared (RMS) measurements and figures were generated using Pymol.
(TIF)

S1 Table Quantitation of temsirolimus levels by HPLC/MS-MS in cell lysates following sucrose density gradient centrifugation and fractionation. (TIF)
