# Supplementary figures and images for: Activation of the unfolded protein response in sarcoma cells treated with rapamycin or temsirolimus
Source: PLoS One. 2017 Sep 19;12(9):e0185089. doi: 10.1371/journal.pone.0185089 (PMC5605117; doi:10.1371/journal.pone.0185089)

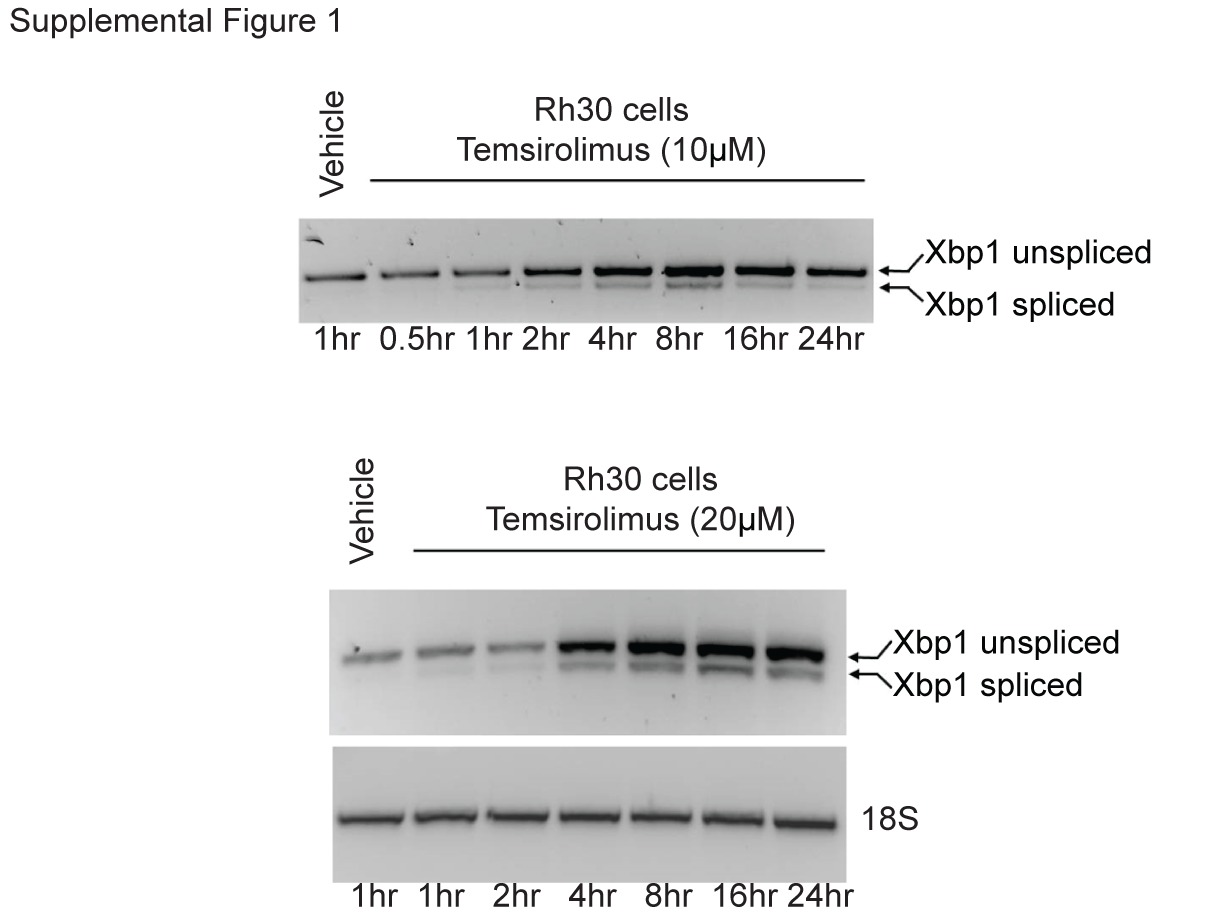

Supplement: S1 Fig — Cells were treated for the indicated times and doses of temsirolimus. Total RNA was harvested and RT-PCR was conducted using a primer pair flanking the unconventional splice site of the Xbp-1 mRNA. PCR amplicons were separated on an agarose gel stained with Gelstar reagent and visualized on a UV transilluminator equipped with a CCD camera. (TIF) [file pone.0185089.s001.tif]

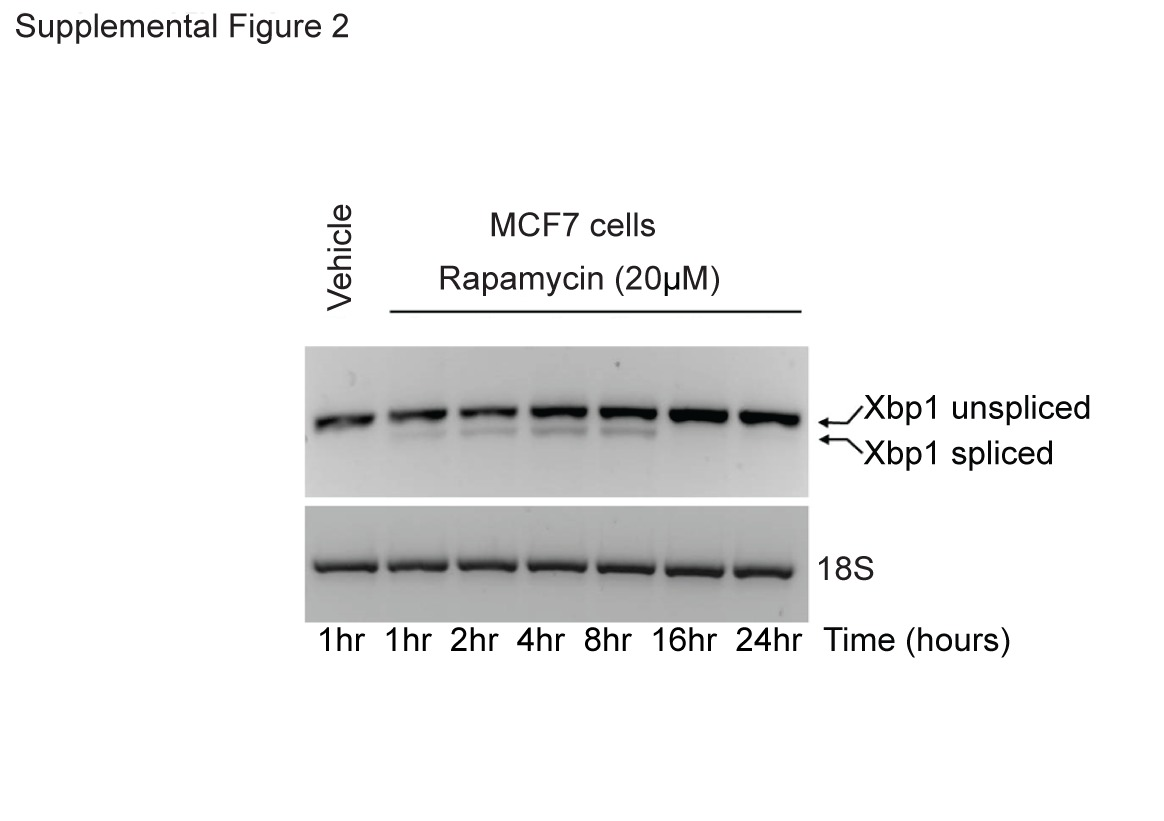

Supplement: S2 Fig — Cells were treated for the indicated times and dose of rapamycin (sirolimus). Total RNA was harvested and RT-PCR was conducted using a primer pair flanking the unconventional splice site of the Xbp-1 mRNA. PCR amplicons were separated on an agarose gel stained with Gelstar reagent and visualized on a UV transilluminator equipped with a CCD camera. (TIF) [file pone.0185089.s002.tif]

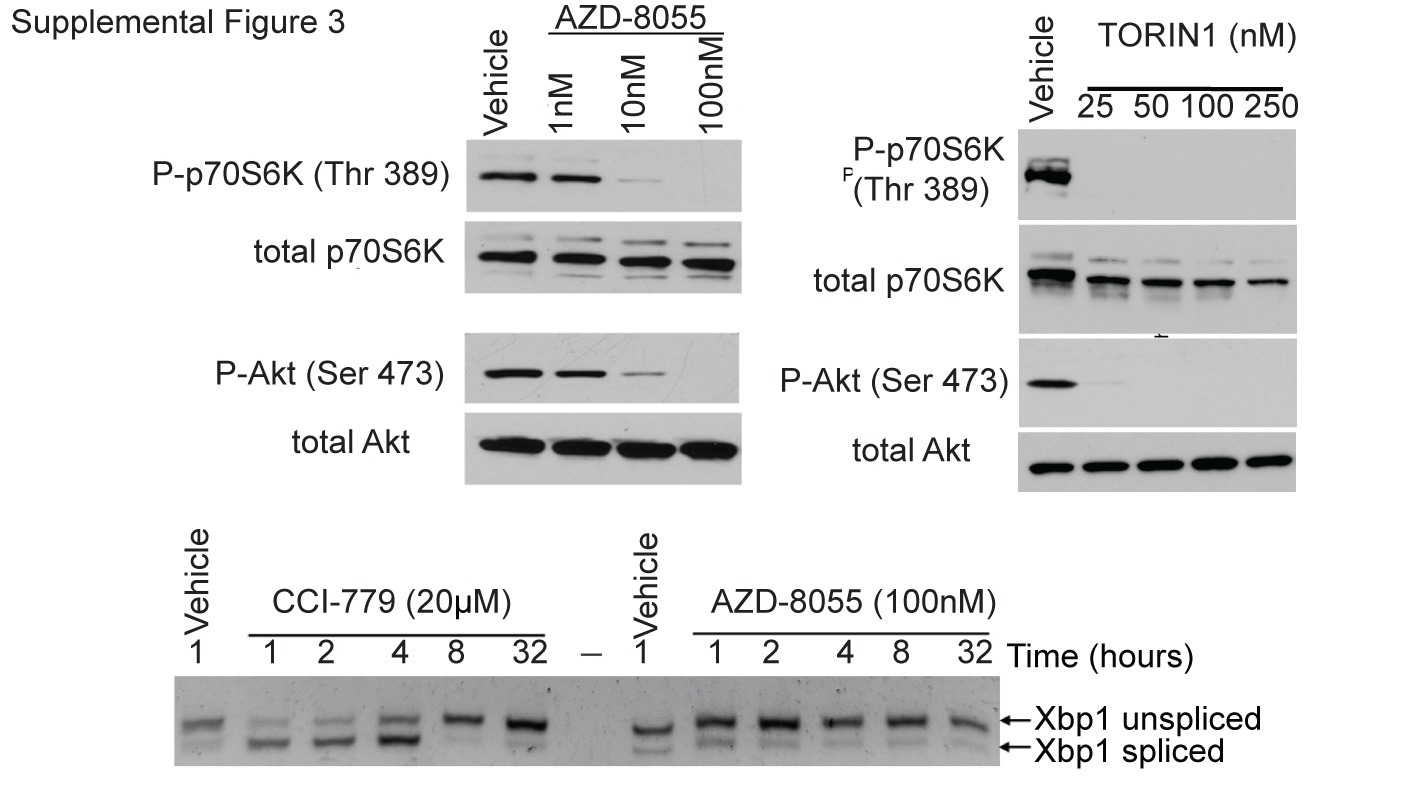

Supplement: S3 Fig — Human 143B osteosarcoma cells were treated for the indicated times and doses of AZD-8055 or Torin1. Total protein was harvested and analyzed by Western blot (upper panels) or total RNA analyzed by RT-PCR (bottom panel) as described in Materials and Methods. (TIF) [file pone.0185089.s003.tif]

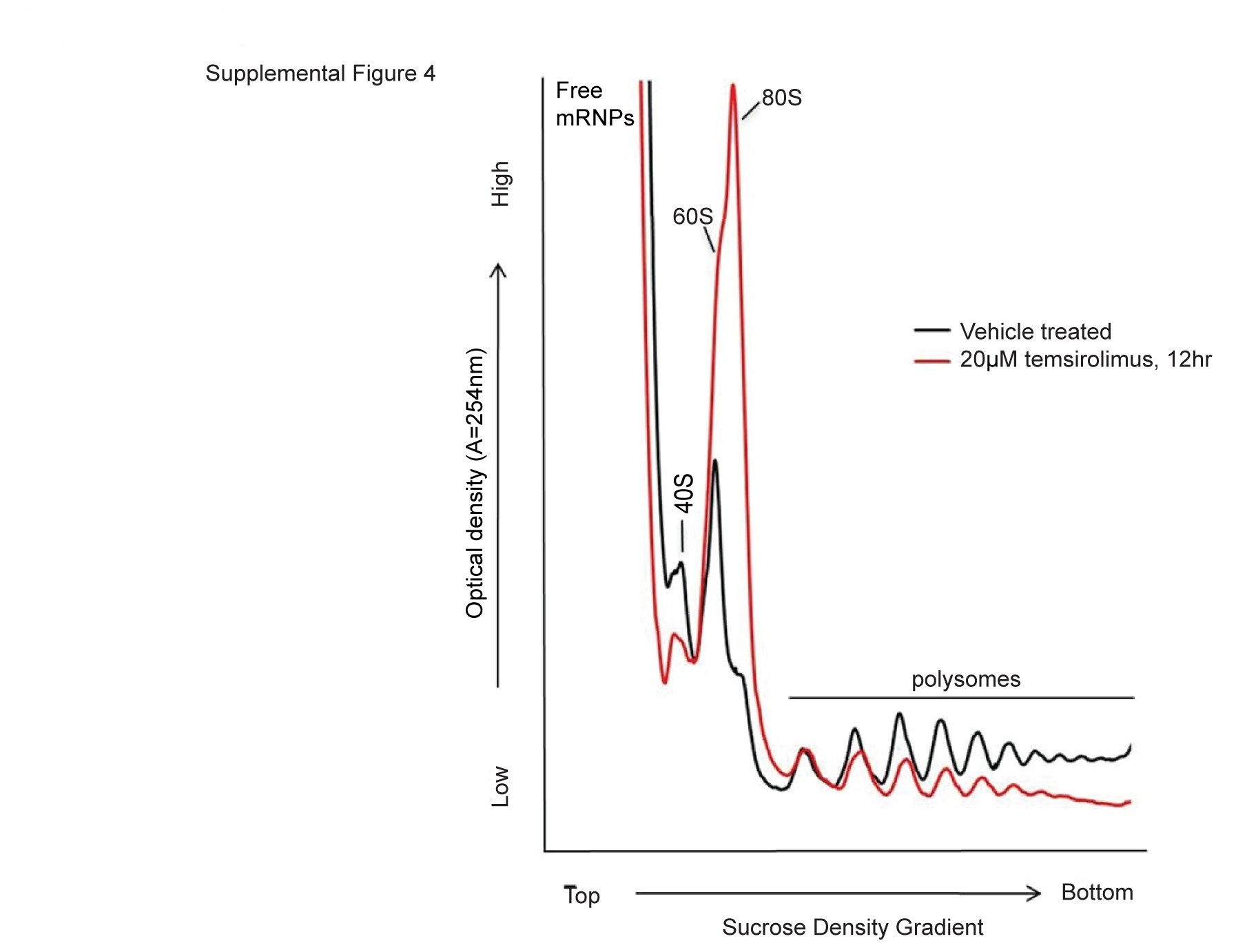

Supplement: S4 Fig — Human Rh30 rhabdomyosarcoma cells were treated for 12hrs with the indicated dose of temsirolimus. Post-mitochondrial supernatant was layered on 15–45% sucrose density gradients and fractionated as described in Materials and Methods. The location of free mRNPs, 40S and 60S ribosomal subunits, 80S monosomes and polysomes are noted. The optical density (A = 254nm) was monitored in real-time and plotted along the y-axis. Gradient depth is plotted along the x-axis. (TIF) [file pone.0185089.s004.tif]

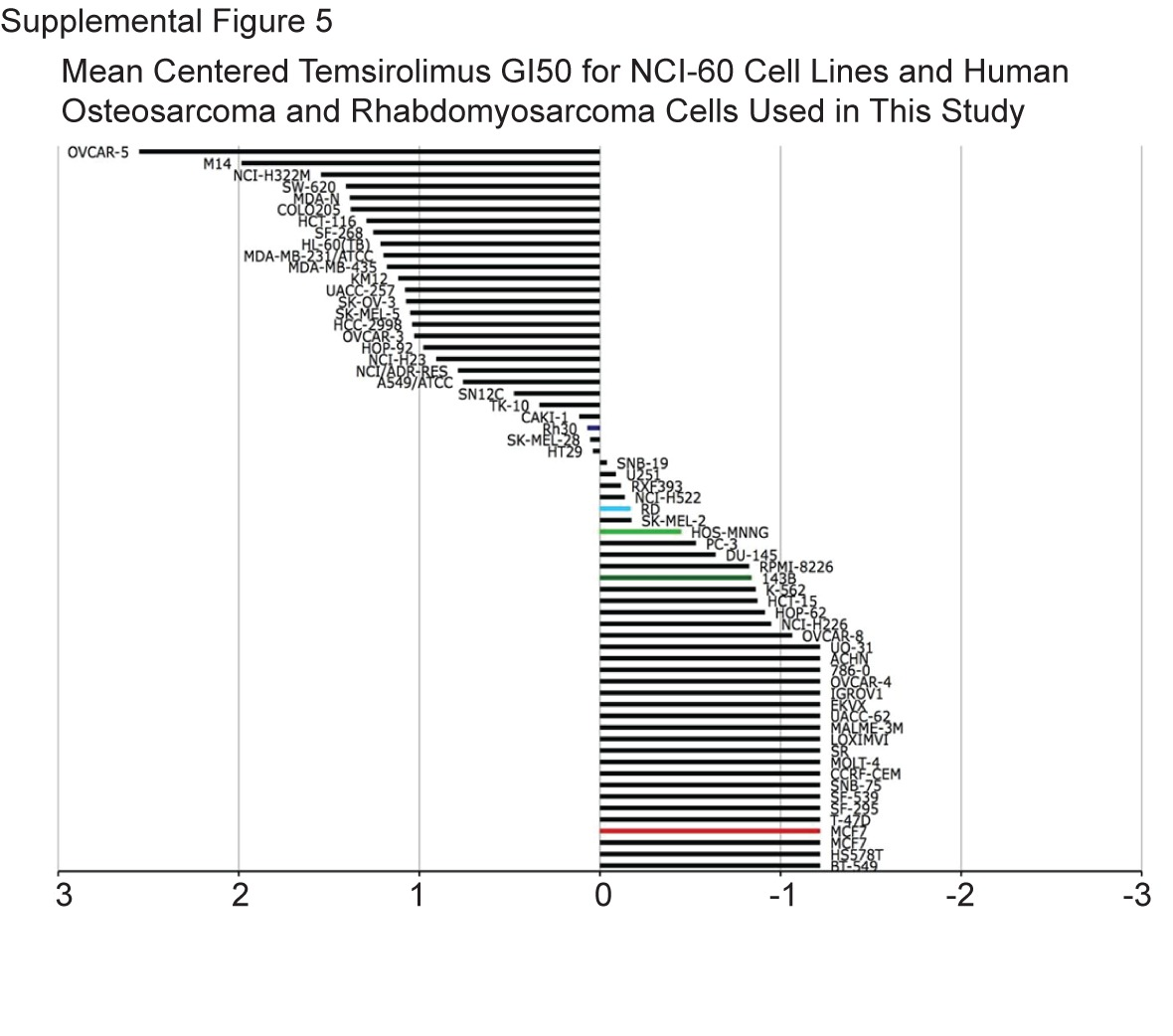

Supplement: S5 Fig — Data for the NCI-60 cell lines was obtained from the Developmental Therapeutics Program (DTP) at the National Cancer Institute. Cell lines used in our current study (143B, Rh30, RD, MCF7) were assayed using the same assay and methodology as published for the NCI-60 panel [63]. Values for all cell lines were mean centered and plotted in order to demonstrate that the cells used for this study were not uniquely sensitive or resistant compared to other tumor cell lines. (TIF) [file pone.0185089.s005.tif]

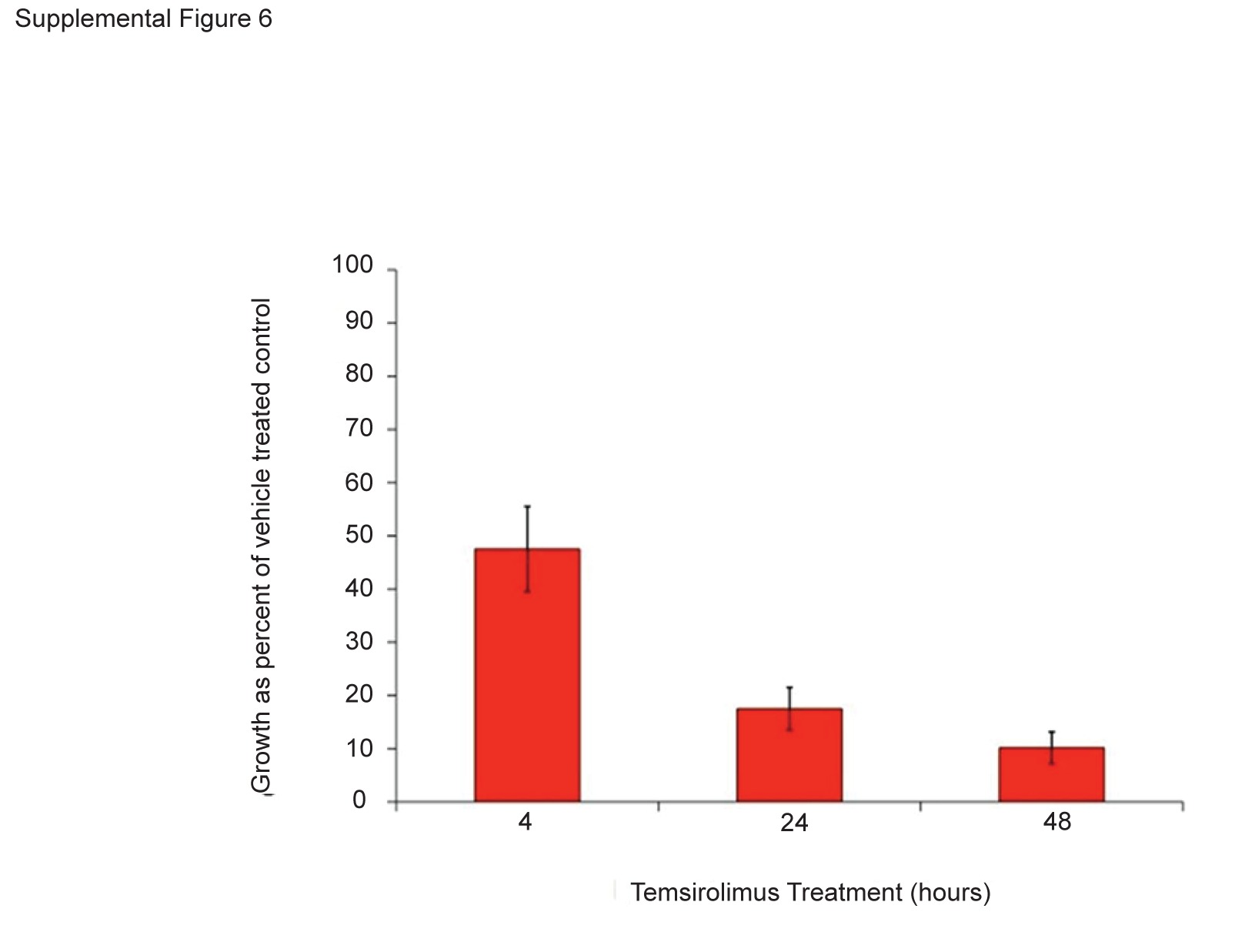

Supplement: S6 Fig — Human 143B OS cells were treated for the indicated durations with 20μM temsirolimus and assayed for growth, compared to vehicle treated, at 48hrs. For example, cells were exposed to 20μM temsirolimus for 4hrs, extensively washed to remove drug and then refed with growth medium in the absence of drug for another 44hrs. Samples were then compared to vehicle treated using the sulforhodamine B assay as described in Materials and Methods. (TIF) [file pone.0185089.s006.tif]

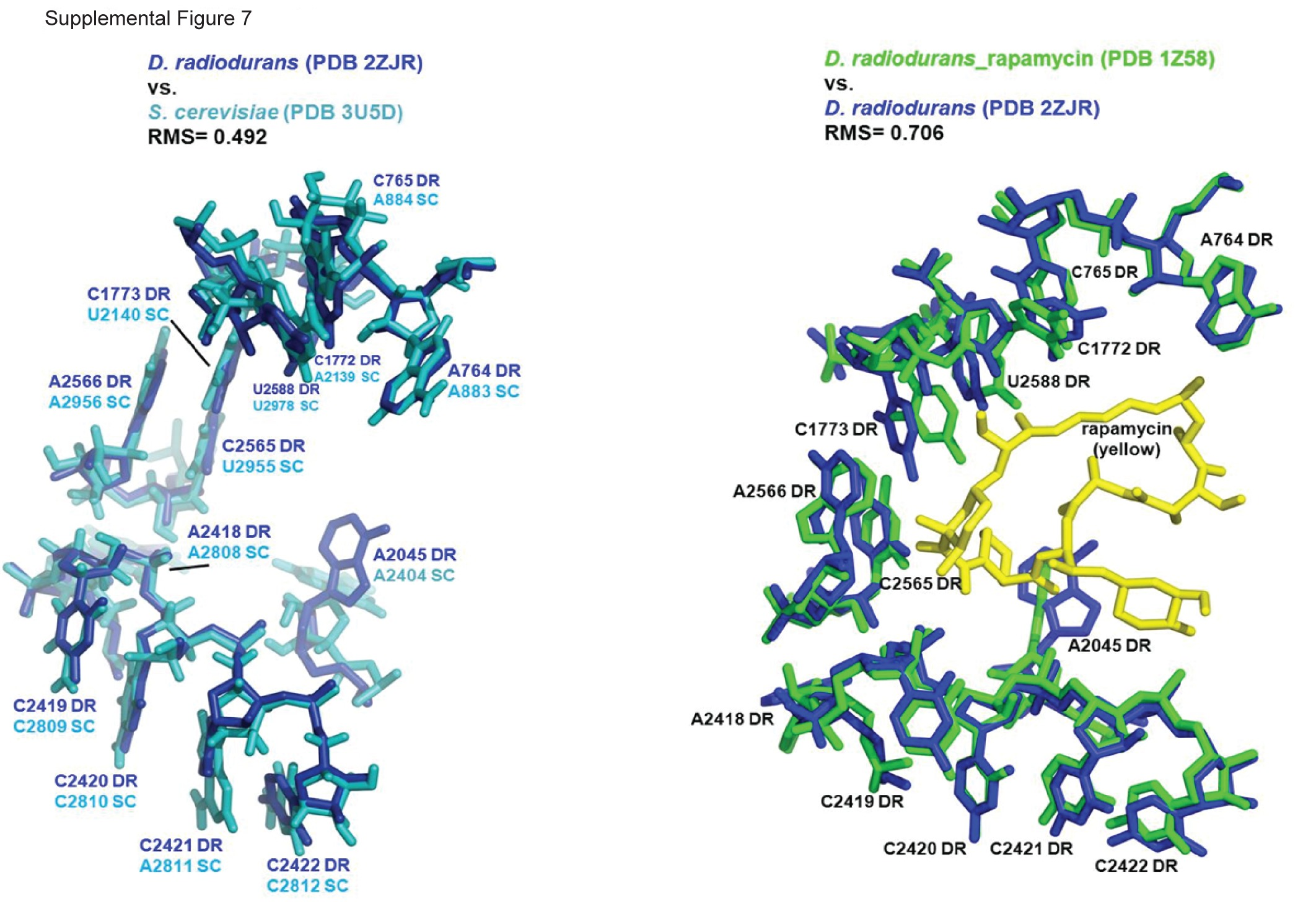

Supplement: S7 Fig — The left panel is the result of alignment between D. radiodurans (dark blue) in the native state without rapamycin bound and S. cerevisiae (cyan) [54, 66]. The right panel is the result of an alignment between rapamycin bound (green) and unbound (blue) D. radiodurans X-ray crystal structures [52, 66]. All alignments, root-mean squared (RMS) measurements and figures were generated using Pymol. (TIF) [file pone.0185089.s007.tif]

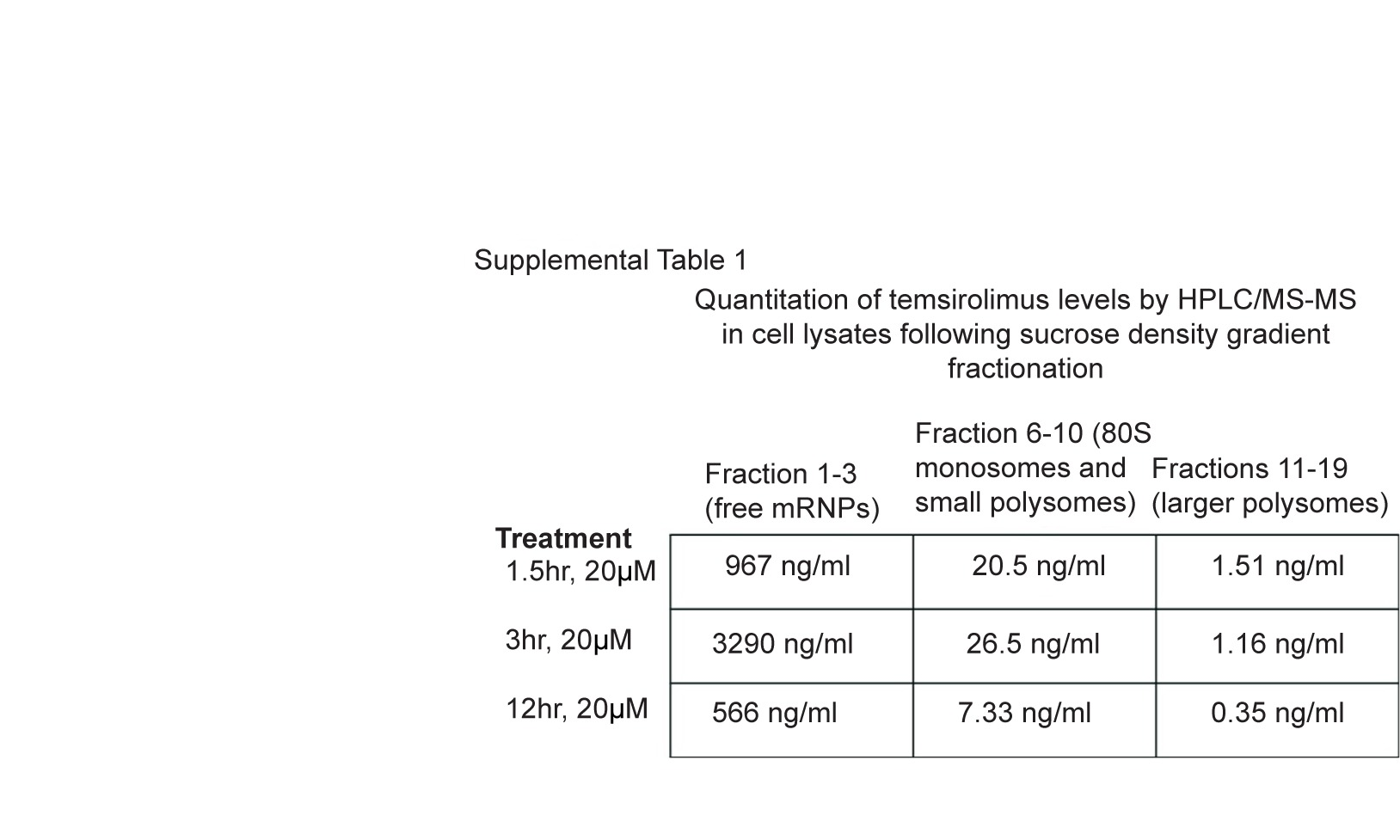

Supplement: S1 Table — (TIF) [file pone.0185089.s008.tif]
